# Novel Analgesic Index for Postoperative Pain Assessment Based on a Photoplethysmographic Spectrogram and Convolutional Neural Network: Observational Study

**DOI:** 10.2196/23920

**Published:** 2021-02-03

**Authors:** Byung-Moon Choi, Ji Yeon Yim, Hangsik Shin, Gyujeong Noh

**Affiliations:** 1 Department of Anaesthesiology and Pain Medicine Asan Medical Center University of Ulsan College of Medicine Seoul Republic of Korea; 2 Department of Biomedical Engineering Chonnam National University Yeosu Republic of Korea; 3 Department of Clinical Pharmacology and Therapeutics Asan Medical Center University of Ulsan College of Medicine Seoul Republic of Korea

**Keywords:** analgesic index, machine learning, pain assessment, photoplethysmogram, postoperative pain, spectrogram

## Abstract

**Background:**

Although commercially available analgesic indices based on biosignal processing have been used to quantify nociception during general anesthesia, their performance is low in conscious patients. Therefore, there is a need to develop a new analgesic index with improved performance to quantify postoperative pain in conscious patients.

**Objective:**

This study aimed to develop a new analgesic index using photoplethysmogram (PPG) spectrograms and a convolutional neural network (CNN) to objectively assess pain in conscious patients.

**Methods:**

PPGs were obtained from a group of surgical patients for 6 minutes both in the absence (preoperatively) and in the presence (postoperatively) of pain. Then, the PPG data of the latter 5 minutes were used for analysis. Based on the PPGs and a CNN, we developed a spectrogram–CNN index for pain assessment. The area under the curve (AUC) of the receiver-operating characteristic curve was measured to evaluate the performance of the 2 indices.

**Results:**

PPGs from 100 patients were used to develop the spectrogram–CNN index. When there was pain, the mean (95% CI) spectrogram–CNN index value increased significantly—baseline: 28.5 (24.2-30.7) versus recovery area: 65.7 (60.5-68.3); *P*<.01. The AUC and balanced accuracy were 0.76 and 71.4%, respectively. The spectrogram–CNN index cutoff value for detecting pain was 48, with a sensitivity of 68.3% and specificity of 73.8%.

**Conclusions:**

Although there were limitations to the study design, we confirmed that the spectrogram–CNN index can efficiently detect postoperative pain in conscious patients. Further studies are required to assess the spectrogram–CNN index’s feasibility and prevent overfitting to various populations, including patients under general anesthesia.

**Trial Registration:**

Clinical Research Information Service KCT0002080; https://cris.nih.go.kr/cris/search/search_result_st01.jsp?seq=6638

## Introduction

Efficient management of postoperative pain affecting the prognosis of patients is becoming increasingly important [1]. To properly administer analgesics, it is necessary to first objectively assess the patient’s degree of pain. In conscious patients, pain can be assessed by asking the patient directly, but unconscious patients or those with difficulty communicating require an appropriate index to quantify their pain. However, current commercial analgesic indices were developed for the purpose of evaluating nociception in patients under general anesthesia [2,3]; therefore, there is no standard for the quantification of postoperative pain in conscious patients [4]. Thus, developing a new pain index to quantify pain in patients who cannot directly communicate their level of pain may also help in the clinical setting; it will also reduce the need to ask questions each time the patient is conscious when pain must be evaluated frequently.

A photoplethysmogram (PPG) is a biosignal that can be obtained continuously and noninvasively using a pulse oximeter. Because a PPG conveys much information about a patient’s condition, many attempts have been made to quantify pain by analyzing PPG signals [3,5-7]. The surgical pleth index (SPI; GE Healthcare), developed for quantifying nociception during general anesthesia, only considers the amplitude and heartbeat interval of a PPG [3]. In addition to these 2 parameters, other pain-related features are present in PPG signals [6,7]. Therefore, the application of a new analytical method has the potential to improve the performance of analgesic indices.

Deep learning architectures, such as a convolution neural network (CNN), can be a good solution to elucidate the hidden features in a PPG because they can identify optimal abstracted features that are beyond human comprehension without any manual procedure [8]. Furthermore, in determining the presence of pain, machine learning has a strong advantage owing to its nonlinear characteristics compared with the SPI, which assesses pain based on simple linear regression [8,9], potentially making it possible to effectively predict nonlinear deviations among individuals or situations [10]. Therefore, a combination of the extended features of PPG and machine learning–based scoring is expected to overcome the limitations of existing pain assessment techniques. However, because a PPG is a 1D signal, whereas CNNs have the advantage of multidimensional data analysis, a dimensional extension of a PPG without loss of time–frequency characteristics is required to apply it optimally in a CNN. A spectrogram, which is a 2D image including the intact time–frequency characteristics of a PPG, can be a good method for applying a CNN to PPGs.

This study aimed to develop a new analgesic index using PPG spectrograms and a CNN to objectively assess pain in conscious patients. In addition, the performance of our newly developed index was compared with that of the SPI.

## Methods

### Patient Population

The study protocol was approved by the Institutional Review Board of Asan Medical Centre (approval number: 2016-0477) and registered on an international clinical trials registry platform (registration number KCT0002080). Written informed consent was obtained from all patients. All procedures were conducted in accordance with relevant guidelines and regulations. In total, 120 patients (American Society of Anesthesiologists Physical Status 1, 2, or 3) between the ages of 20 and 80, who were scheduled to undergo elective surgery, were included in this observational study. Exclusion criteria were as follows: clinically significant impairment of the cardiovascular, hepatic, or renal function; history of cardiac arrhythmia; use of medication that might affect autonomic function; the presence of presurgical acute or chronic pain (Visual Analog Scale score [VAS] > 0, measured before surgery); clinically significant laboratory findings; and evidence of pregnancy.

### Procedure and Data Acquisition

All patients fasted from midnight on the day of surgery without premedication. In the operating theater, patients were monitored for their heart activity using electrocardiography, end-tidal carbon dioxide partial pressure, and noninvasive blood pressure measurement. Neuromuscular transmission was monitored using an M-NMT module at the adductor pollicis muscle (CARESCAPE B850; GE Healthcare). A reusable SPI sensor was placed on the index finger of each patient (on the arm not used for blood pressure measurement). Patients were allowed to acclimatize for at least 5 minutes in the supine position in a quiet operating theater, after which baseline data (without pain) were collected for 6 minutes, of which the latter 5 minutes were used for analysis. General anesthesia was performed by administering propofol and remifentanil by a target effect-site concentration–controlled infusion using the Schnider and Minto models [11,12]. Target effect-site concentrations (*Ces*) of propofol were titrated to maintain the bispectral index (Covidien) at less than 60 during the induction and maintenance of anesthesia. The target *Ces* of remifentanil were adjusted to maintain stable hemodynamics (ie, systolic blood pressure >80 mmHg and heart rate over 45 beats/min). All patients received a bolus dose of oxycodone (0.1 mg/kg) 30 minutes prior to the end of surgery.

Intravenous patient-controlled analgesia with oxycodone began after the administration of the bolus dose of oxycodone. Neuromuscular blockade was reversed with neostigmine and glycopyrrolate at the end of surgery. Tracheal extubation was performed when the train-of-four ratio was greater than 0.9 and bispectral index value was greater than 80. Patients were then transported to the postanesthesia care unit (PACU). When the patients arrived in the PACU, their state of consciousness was assessed with a modified Aldrete score [13]. Electrocardiogram, pulse oximetry, and noninvasive blood pressure were also monitored. Additional PPG and SPI data were obtained for the initial 5 minutes in the PACU. After obtaining the data, patients were assessed for pain using a VAS (0=no pain; 100=the most severe pain). Oxycodone was administered according to postoperative pain intensity. The PPG and SPI values were measured using an S/5 Anesthesia Monitor (Datex-Ohmeda, Inc.) and recorded on a laptop for offline analysis. The PPG data were sampled at 300 Hz, and SPI data were recorded every 10 seconds.

### Pain Assessment Model

A spectrogram–CNN model was developed and validated through fivefold cross-validation. The developed model outputs the spectrogram–CNN index as a pain score using a PPG spectrogram as input and CNN as a pain scorer. During model development, patients and test sets were separated to prevent intrasubject interference to avoid data overlaps between the development and test sets of each fold. For model training, 90% of the development set was used as a training set and 10% as a validation set. Finally, for each fold, 20, 73, and 7 patients’ data were used as a test set, training set, and validation set, respectively. Pain and nonpain data labels were created based on the VAS, where VAS>0 was defined as pain and VAS=0 was defined as nonpain with labels “1” and “0,” respectively. Detailed descriptions of the spectrograms and CNN used in this study are given below.

#### Spectrogram

**Figure 1:**
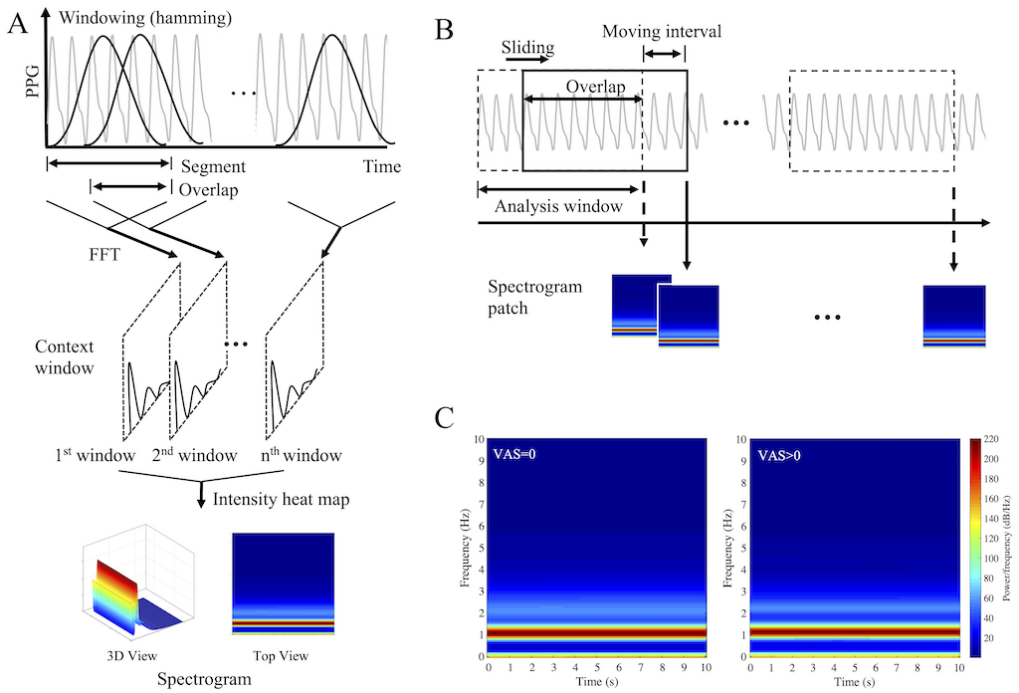
Process for generation of a single spectrogram (A), process of generating multiple spectrograms over time using a sliding window (B) and the average spectrogram during pain (left) and non-pain (right) conditions (C); FFT: fast Fourier transform; PPG: photoplethysmogram.

Spectrogram creation is a method used for time–frequency analysis of time series signals. A spectrogram reconstructs 2D images while maintaining the information contained in 1D time series data [14]. Spectrograms are useful for visually describing changes in the frequency characteristics of nonstationary signals, such as physiological signals over time [15,16]. Spectrograms can be generated by repeating short-time Fourier transforms that divide a longer time signal into shorter segments of equal length and then computing the Fourier transforms separately on each shorter segment. In this study, 2D spectrogram images generated from 1D PPGs were used as the pain classifier input to reflect the whole waveform and change of waveform, not the specific feature of the PPG waveform. Prior to spectrogram generation, all PPGs were filtered using both a finite impulse response bandpass filter with a 0.5-10-Hz passband and a 30-tap moving average filter. In addition, considering that PPG amplitude is an arbitrary unit, a normalization process was performed to reduce the intersubject and intermeasurement deviations [17]. In the normalization process, *z*-scores were obtained by subtracting the mean of the measured values from each measured value and dividing by the standard deviation. The spectrogram generation process is illustrated in Figure 1.

Spectrogram images were generated by short-time Fourier transforms of 10-second PPGs every 10 seconds without overlap (Figure 1A). At the time, to generate a single spectrogram image, each 10-second PPG was divided into 6.3-second segments with a 6.27-second overlap and transformed to the frequency domain using fast Fourier transform after windowing with a Hamming window (Figure 1B). The frequency range of the spectrogram image was set to 0-10 Hz, and the frequency resolution was set to 0.81 Hz to equalize the number of time frames and frequency bins, that is, to equalize the numbers of horizontal and vertical pixels in the spectrogram image, respectively. Finally, 30 spectrogram patches of size 124 × 124 were generated for each 5-minute PPG. Figure 1C shows the averaged spectrograms without pain (left) and with pain (right). All preprocessing and spectrogram patches were generated using MATLAB (version 2018a; The MathWorks, Inc.).

#### Convolutional Neural Network

**Figure 2:**
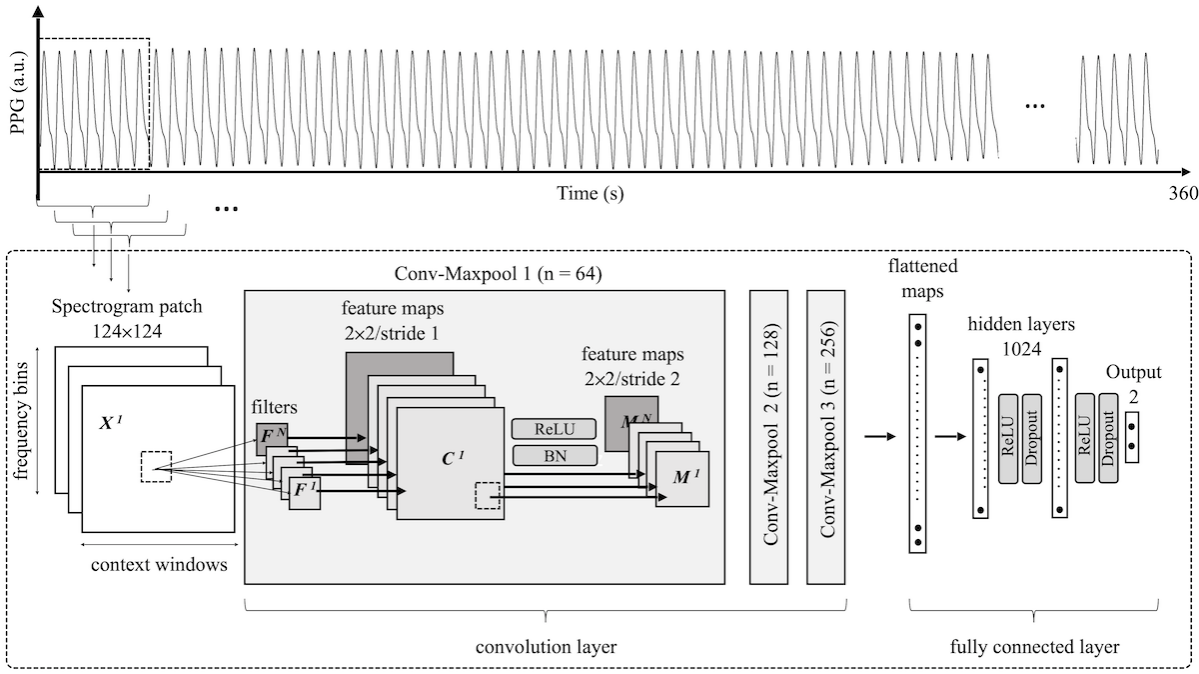
Architecture of the convolutional neural network proposed in this study. X: input, F: filter, C: convolution layer, M: max pooling, N: number of filters, BN: batch normalisation, ReLU: rectifier linear unit, Conv-Maxpool: convolution-max pooling.

CNNs are useful for image analysis because they have the advantage of maintaining the spatial information of 2D or higher inputs. CNNs enable data-driven learning, are highly representative, and effectively combine the spatial information of multidimensional inputs [18]; supervised CNNs extract information more effectively due to class-specific information [19,20]. The CNN used in this study has a 2D PPG spectrogram input and binary-coded labels: “0” is a pain-free state and “1” is pain state. Figure 2 shows the structure of the CNN developed in this study. First, in the convolution-max pooling layer (Conv-Maxpool), 32, 64, and 128 filters are applied to the spectrogram input to perform a convolution process, and the spatial characteristics are simplified through max pooling. In Conv-Maxpool, the filter size of all convolutional layers is 2 × 2 and the stride is 1. The size of the feature map is reduced by setting the filter size of the max pooling layer to 2 × 2 and stride to 2, equal to the filter size. A batch normalization layer and rectified linear unit (ReLU) activation functions are applied to the Conv-Maxpool to increase learning speed and efficiency. Batch normalization is a structure that improves the speed and stability of neural networks by normalizing interlayer input data [21], and ReLU improves the expressive power of neural networks based on nonlinear features [22,23]. The Conv-Maxpool process is repeated 3 times, resulting in a feature map size of 15 × 15. The fully connected layer consists of 2 hidden layers and 1 output layer, and the ReLU activation function and dropout are also applied to the hidden layer to reduce overfitting [24]. The dropout rate was set to 0.5 in training, but there was no dropout during testing. Cross-entropy is employed as a cost function [25], and adaptive moment estimation (Adam) is used as an optimizer [26]. Finally, the result is output to 2 nodes, representing “pain free” and “pain,” and the values are probabilistically expressed using the SoftMax function [27]. Because the output of SoftMax gives the probability of the input data being a painful condition with a value between 0 and 1, it is converted into a pain index as the “likelihood of pain.” Consequently, the spectrogram–CNN index is calculated by multiplying the probability value output from the pain node by 100. The CNN model was implemented and trained using Python 3.7 (Python Software Foundation) and TensorFlow 2.0 in the Anaconda environment.

### Statistical Analyses

Receiver-operating characteristic (ROC) curves were computed to compare the sensitivity and specificity of the spectrogram–CNN index and SPI for detecting pain. Cutoff values used for calculation of sensitivity and specificity were computed as “best fit” (highest combined sensitivity and specificity) [28]. The differences between the spectrogram–CNN index and SPI in the ROC curves were calculated using the MedCalc Statistical Software (version 13.3.1; MedCalc Software). Statistical analyses were conducted using IBM SPSS (version 22.0; SPSS Inc.). Data are expressed as mean (SD) for normally distributed continuous variables, median (25%-75%) for non-normally distributed continuous variables, and count SPI for categorical variables. *P* values <.05 were considered to be statistically significant.

## Results

In total, 120 patients were enrolled, of whom 20 dropped out because of failure of PPG data storage (n=8), failure of SPI data storage (n=2), abnormal SPI data that could not be included in the analysis (n=9), and failure to measure VAS after surgery (n=1). Thus, 100 patients were included in the final analysis. The characteristics of these patients are summarized in Table 1. All but 1 patient had consciousness values of 2 points (conscious), as assessed by the modified Aldrete score at the time of PACU arrival. One patient scored 1 (arousable on calling) who later had a value of 2 points upon leaving the PACU.

**Figure 3:**
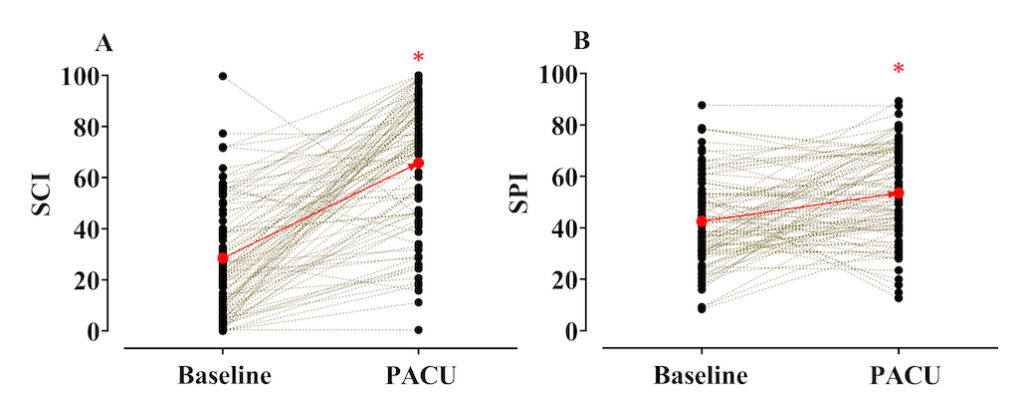
Individual changes (n=100) in the spectrogram-convolutional neural network index (SCI, A) and the Surgical Pleth Index (SPI, B) and without and with pain. *P<.05 vs. baseline. The black circles represent the average of 5 min of the SCI or SPI observed without and with pain. The red circles indicate mean values for all patients. PACU: postanaesthesia care unit.

**Figure 4:**
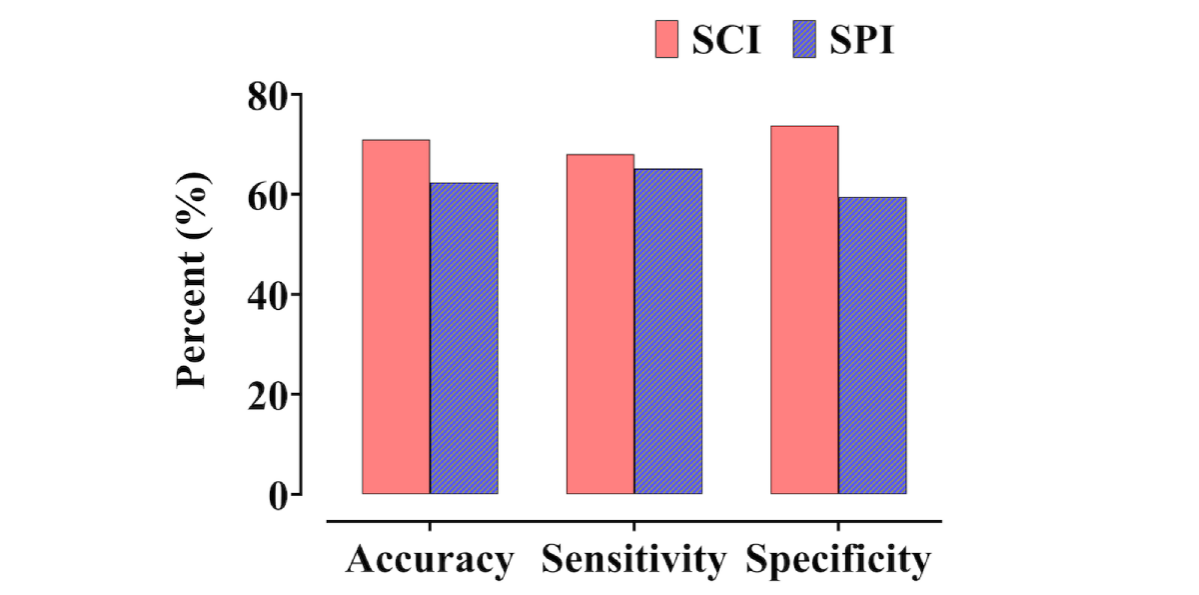
Performance measures of spectrogram-convolutional neural network index (SCI, red) and the Surgical Pleth Index (SPI, blue) in terms of accuracy, sensitivity and specificity. Accuracy means balanced accuracy. Cut-off value of SCI was 48.

Individual changes in the spectrogram–CNN index and SPI without and with pain are presented in Figure 3. The 7 patients who had no postoperative pain were excluded from this analysis. In the case of pain, the mean spectrogram–CNN index and SPI values increased significantly (baseline spectrogram–CNN index: 28.5 (SD 22.1) versus PACU spectrogram–CNN index: 65.7 (SD 25.4), *P*<.01 in paired *t* test; baseline SPI: 42.5 (SD 16.7) versus PACU SPI: 53.5 (SD 17.8), *P*<.01 in paired *t* test). The area under the curve (AUC) of the ROC and cutoff values for detecting pain in terms of spectrogram–CNN index and SPI are listed in Table 2. The spectrogram–CNN index was statistically superior to the SPI (pairwise comparison of ROC curves: spectrogram–CNN index versus SPI, *P*<.01). Moreover, as shown in Table 2 and Figure 4, the spectrogram–CNN index showed improved performance measures in terms of balanced accuracy, sensitivity, and especially specificity.

**Table 1:** Characteristics of the study population.

Characteristic		Patients (N=100)
Male/Female		44/56
Age (years), mean (SD)		53.4 (12.5)
Height (cm), mean (SD)		161.8 (8.5)
Weight (kg), mean (SD)		62.2 (12.2)
ASA PS^a^ 1/2/3		22/76/2
**Type of surgery, n**		
	Breast	29
	Colorectal	19
	Hepatobiliary	12
	Stomach	30
	Thyroid	10
**Postoperative pain intensity at PACU^b^, n**		
	No (VAS^c^=0)	7
	Mild (0 < VAS ≤ 30)	11
	Moderate (30 < VAS ≤ 70)	58
	Severe (70 < VAS ≤ 100)	24

^a^ASA PS: American Society of Anesthesiologists Physical Status.

^b^PACU: postanesthesia care unit.

^c^VAS: Visual Analog Scale (0=no pain; 100=the most severe pain).

**Table 2:** Areas under the receiver-operating characteristic curves (AUCs) and cutoff values for assessing pain in the spectrogram-convolutional neural network index and surgical pleth index (SPI) in surgical patients.

Parameter	Spectrogram–CNN^a^ index			SPI
	Training set	Validation set	Test set	
AUC (95% CI)	0.992 (0.991-0.993)	0.932 (0.921-0.942)	0.757 (0.746-0.768)	0.659 (0.646-0.671)
*P* value	<.01	<.01	<.01	<.01
Cutoff value^b^	50	54	48	44
Sensitivity, %, specificity, %	94.6, 96.4	83.1, 88.2	68.1, 73.8	65.2, 59.5
Balanced accuracy, %^c^	95.5	85.7	71.0	62.4

^a^CNN: convolutional neural network.

^b^Cutoff values used for the calculation of sensitivity and specificity were calculated as “best fit” (highest combined sensitivity and specificity).

^c^Balanced accuracy is the corrected accuracy of the imbalance of a class set, calculated as (sensitivity + specificity)/2.

**Figure 5:**
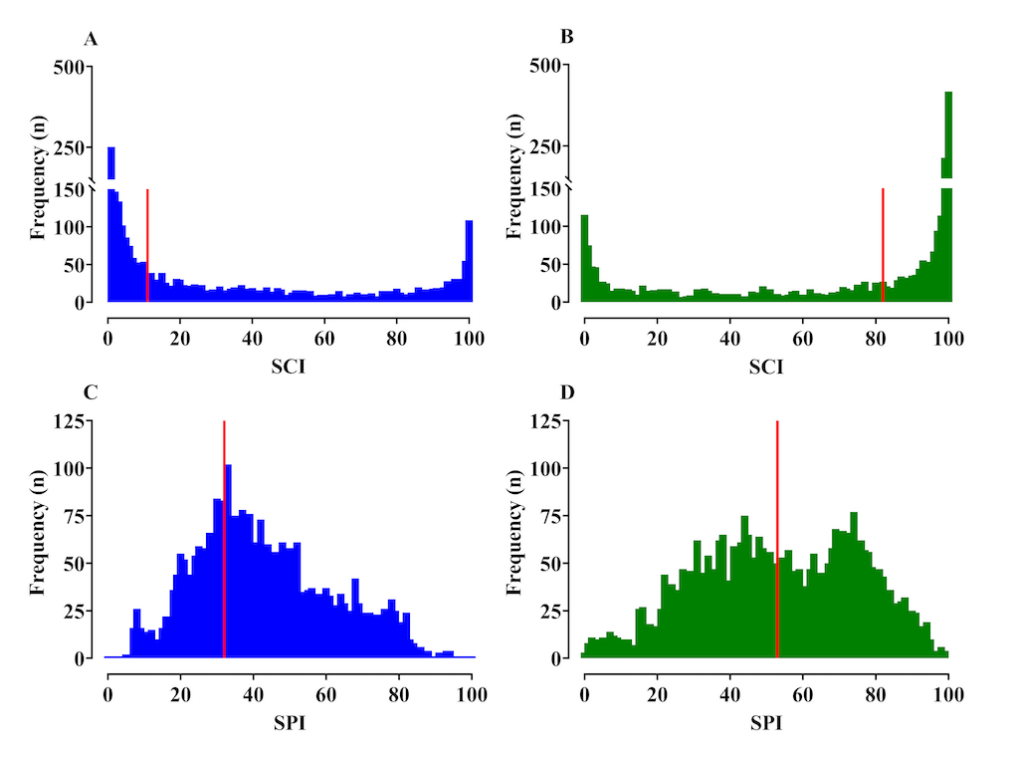
Frequency distribution of the spectrogram-convolutional neural network index (SCI, A and B) and surgical pleth index (SPI, C and D) values observed without (A and C) and with (B and D) pain. During the data collection period (baseline: 5 min, postanaesthesia care unit: 5 min), the SCI and SPI values were observed every 10 sec. The vertical red lines show the median frequency (A: 11, B: 82, C: 32, D: 53).

Neither spectrogram–CNN index (mild: 69.5 [53.2-87.7], moderate: 71.8 [40.5-89.3], severe: 74.6 [62.9-84.7], *P*=.78, Kruskal–Wallis one-way ANOVA on ranks) nor SPI (mild: 53.5 [SD 21.0], moderate: 51.8 [SD 18.0], severe: 57.9 [SD 15.7], *P*=.37; one-way ANOVA) could statistically distinguish between mild, moderate, and severe pain. The frequency distributions of the spectrogram–CNN index and SPI values observed without and with pain during the data collection period are shown in Figure 5. The distribution of SPI values overlapped for with and without pain, suggesting that the SPI shows several false positives/false negatives, whereas the distribution of spectrogram–CNN index values showed a significant difference (*P*<.05) in patients with and without pain, suggesting that the spectrogram–CNN index can distinguish pain more clearly than the SPI.

## Discussion

The spectrogram–CNN index proposed in this study outperformed the commercialized SPI pain index in the postoperative pain assessment of conscious patients. One of the main reasons for its outperformance is the spectrogram input containing the PPG’s intact waveform information, making it possible to use hidden pain-related factors that could not be provided only by the peaks. Because existing pain assessment methods, such as the SPI, use only certain features of a PPG that show significant changes in surgical stimuli, numerous pain-related information reflected in the PPG may be overlooked. To overcome these limitations, research has been conducted on new pain-related features derived from sophisticated PPG waveform analysis in addition to the heart rate interval and PPG amplitude reflected in the SPI [5-7]. However, these pain indicators still depend on complex processes, such as peak detection and feature extraction, which require accurate peak extraction algorithms and are vulnerable to signal quality degradation. The proposed PPG spectrogram input does not require any peak detection or feature extraction process, thus avoiding problems such as peak misdetection during preprocessing. In addition, the spectrogram provides information from almost the whole PPG waveform because only the domain in which information is represented is transformed, while the underlying information is retained. This feature of the proposed model is its differentiating factor from SPI, which requires a complicated process of extracting pain features from biosignals, including PPG pulsation start and systolic maximum detection and verification. These preprocessing steps are necessary in existing pain assessment methods, but they are cumbersome and vulnerable, providing only limited features. Therefore, a simplified preprocessing process that still provides plentiful feature information can significantly improve the robustness of pain assessment.

Another key technique proposed in this study is the discrimination of postoperative pain using machine learning. Although machine learning–based pain assessment has already been studied [29-31], it is not suitable for practical clinical situations because it depends on high-dimensional clinical data, such as patient records and electroencephalograms, which are rarely used in postoperative care. However, machine learning from PPGs, which are frequently used in clinical practice during postoperative care, has high practical utility. In this study, we used a spectrogram–CNN combination, which has already been applied to electrocardiograms and electroencephalograms and has shown reasonable performance in predicting seizures and atrial fibrillation [32-35]. The spectrogram converts the data into 2D, and the CNN has the advantage of extracting the spatial features of multidimensional data. The combination of these techniques thus extends the dimensions of PPG and allows spatiotemporal analysis, maximizing the use of features inherent in the signal. Nonlinear classification may be another important reason for the good performance of the proposed model. While the SPI is derived from a simple linear combination of normalized heartbeat interval and normalized pulse wave amplitude [3,36], the proposed spectrogram–CNN model performs nonlinear classification using the ReLU activation function. In addition, the nonlinearity is increased because the ReLUs are overlapped with each other in a multilayered structure.

In this study, patient data used for model development and validation were separated, and fivefold cross-validation was performed to eliminate interindividual interference and to generalize the model. Therefore, the proposed spectrogram–CNN index is expected to show similar performance in other groups of patients of similar age who did not participate in model development. However, there may be a few new variables to consider. In Table 2, performance measures were approximately 95%, approximately 85%, and approximately 70% for the training, validation, and test sets, respectively. The decision criteria also differed with 50 in the training set, 54 in the validation set, and 48 in the test set, indicating that overfitting occurred. In a random permutation test on balanced accuracy, the average accuracy was 49.5 (SD 0.7), and the range of values was 47.6-51.8. Considering that all balanced accuracies of the training, validation, and test sets were over 71, it is likely that the overfitting in our result stemmed from large intersubject variability rather than the model itself. Overfitting can be interpreted as degradation of the model’s versatility, but it can also be interpreted as higher performance, at least in terms of validation accuracy, if sufficient data are used. Therefore, further studies are required to improve the reliability and versatility of this index using a large patient population with diverse body characteristics.

The spectrogram–CNN index may not have been able to distinguish pain intensity because the number of observations was relatively small. A previous study showed that SPI distinguishes postoperative pain intensity [28]; however, the authors of this previous study analyzed 1300 observations, whereas we only used 93. Nonetheless, a more fundamental reason is that the severity of pain was not accounted for when developing the spectrogram–CNN index. If sufficient PPG data were provided when learning to classify pain severity, it would be possible to classify pain intensity.

The database used in this study was shared by 2 research groups. Another group, independent of this study, has already published their results. Their pain classifier, based on a deep belief network using various PPG features, discriminated well between the presence and absence of pain [37]. Compared to the other study that extracted various features, ours used a simple spectrogram containing all PPG information without any complicated feature extraction procedure and is based on a CNN optimized for the spectrogram input. CNNs have lead in the machine learning field because they demonstrated performance improvements in image recognition in 2012 [9]. Moreover, the previous study evaluated pain based on full-length data, whereas ours assessed pain every 10 seconds, the same data display interval as in the SPI. Therefore, it can be applied to real-time pain assessment, which is in contrast to the other study.

There are some limitations to this study. First, the SPI was selected as a comparative index to evaluate the performance of the spectrogram–CNN index, but its suitability is somewhat debated. The SPI is neither developed nor recommended for use in conscious patients. However, when a new analgesic index is developed, it is essential to evaluate its performance, and this is commonly done by comparing it with an existing index using the same data. Although the SPI is not recommended for use in conscious patients, some studies on conscious patients suggest that SPI can discriminate between the presence and absence of pain [28,38]. Between the SPI and another commonly used commercial analgesic index—the Analgesia Nociception Index (PhysioDoloris, MetroDoloris)—the AUC–ROC for detecting postoperative pain in conscious patients was highest for the SPI [38]. Hence, the SPI was chosen as the comparative index for this study. Second, neostigmine and glycopyrrolate, when administered to reverse neuromuscular blockade, can contribute to PPG signals. As this study was observational, only data necessary for the development of a new analgesic index were collected during the normal anesthesia process without intervention. Neostigmine and glycopyrrolate were used in all patients because none of them required sugammadex. Neostigmine is known to be rapidly eliminated from the plasma after administration, with an average half-life of approximately 25 minutes [39]. We collected postoperative PPG data an average of 29.4 minutes after administration of these 2 agents. A previous study reported that baroreflex sensitivity was restored to its baseline value after approximately 82 minutes of glycopyrrolate administration [40]. It is possible that glycopyrrolate has mixed effects on postoperative PPG. However, because sugammadex usage is not common, it may be more beneficial to develop an index to distinguish pain based on data that can be obtained from actual practical conditions. Further studies are required to evaluate the extent of the effect of these 2 drugs on postoperative PPG. Third, it is difficult to determine whether the PPG data collected from the PACU solely reflect pain. In the conscious state, the sympathetic nervous system may be activated for other reasons, such as arousal or anxiety. Anxiety has been associated with reduced heart rate variability and vagal tone [41]. As we did not evaluate patient anxiety, we cannot determine its contribution to the PPG data. Considering the condition of patients who arrived in the PACU immediately after surgery, the PPG data mostly reflected immediate postoperative pain without controlling consciousness.

In conclusion, although there were several limitations to the study design, we confirmed that the newly developed spectrogram–CNN index can effectively detect postoperative pain in conscious patients. Further validation studies are required to assess its feasibility and prevent overfitting to various populations, including patients under general anesthesia.

## Abbreviations

AUC: area under the curve
CNN: convolutional neural network
PPG: photoplethysmogram
ReLU: rectified linear unit
SPI: surgical pleth index
VAS: Visual Analog Scale
